# Skin Electronics from Biocompatible In Situ Welding Enabled By Intrinsically Sticky Conductors

**DOI:** 10.1002/advs.202202043

**Published:** 2022-06-26

**Authors:** Lixue Tang, Shuaijian Yang, Kuan Zhang, Xingyu Jiang

**Affiliations:** ^1^ School of Biomedical Engineering Beijing Key Laboratory of Fundamental Research on Biomechanics in Clinical Application Capital Medical University No.10 Xitoutiao, You An Men Wai Beijing 100069 China; ^2^ Department of Biomedical Engineering Shenzhen Key Laboratory of Smart Healthcare Engineering, Guangdong Provincial Key Laboratory of Advanced Biomaterials Southern University of Science and Technology No 1088, Xueyuan Rd., Xili, Nanshan District Shenzhen Guangdong 518055 China; ^3^ Beijing Advanced Innovation Center for Big Data‐Based Precision Medicine Capital Medical University Beijing 100069 China

**Keywords:** liquid metals, skin electronics, sticky conductors, stretchable conductors, wearable devices

## Abstract

Welding usually involves high temperatures, toxic solvents, or conditions not compatible with human bodies, which severely limit the fusion of electronics and human tissues. To achieve direct welding of electronics on human skin, the intrinsically sticky conductors that can simultaneously achieve metal‐grade electrical conductivity (≈41 7000 S m^−1^), hydrogel‐grade stretchability (>900% strain), and self‐adhesiveness (1.8 N cm^−1^) are reported. The sticky conductors composed of gallium indium alloy and acrylate polymer adhesives have a surface‐enriched structure, which can form instant mechanical and electrical connections with different surfaces through gentle pressure without involving conditions that may damage human tissues. Based on the sticky conductors, the in situ welding of electronics on the skin is realized. To demonstrate the feasibility of in situ welding, electronic tattoos are achieved for movement monitoring. Intrinsically sticky electrodes that can resist drying and simultaneously deform with the skin for electrophysiological measurement are also developed.

## Graphical abstract

Welding usually involves high temperature and toxic solvent, which limit the fusion of the electronics and human tissues. Herein, the sticky liquid metal conductors that can simultaneously achieve metal‐grade electrical conductivity, hydrogel‐grade stretchability, and self‐adhesiveness are reported. It can form instant connections with different surfaces through gentle pressure without involving conditions that may damage human tissues.

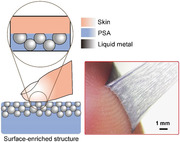

## 1 Introduction

The fusion of soft electronics and human tissues has the potential to improve the quality of human experience in the fields of healthcare monitoring, chronic disease treatment, and human–machine interfaces.^[^ ^1^, ^2^, ^3^, ^4^, ^5^, ^6^, ^7^, ^8^ ^]^ However, the ability to create custom‐made electronics on human organs is hampered by the difficulties in biocompatible in situ welding of electronics on delicate surfaces, such as human skin. At present, most reported skin electronics cannot form a reliable adhesion to the skin, and they usually come into contact with human tissues (such as the skin and the heart) through tapes, bands, or van der Waals forces.^[^ ^9^, ^10^, ^11^, ^12^ ^]^ When the tissue is deformed, the soft electronic device cannot be deformed simultaneously with the tissue due to insufficient adhesion between the device and the tissue, which will result in the detachment of the measurement interfaces and cause measurement errors (e.g., the detachment of the electrode–skin interface will cause huge noise for the electrophysiological measurement).^[^ ^13^, ^14^ ^]^ Some hydrogels are reported to be soft, conductive, and sticky at the same time.^[^ ^15^, ^16^, ^17^, ^18^ ^]^ However, they will lose both electrical conductivity and adhesion after long‐term air exposure until they inevitably dry out. And the electrical conductivity of hydrogels (usually 0.1–10 S m^−1^) is much lower than that of metals. To achieve sticky conductors, some researchers developed intrinsic adhesive conductors^[^ ^19^ ^]^ and conductors with sucker‐shaped microstructures.^[^ ^20^, ^21^ ^]^ However, both the preparation and the patterning process of such conductors are complicated, and it is difficult to use those conductors to fabricate stretchable circuits. Welding rigid electronics on soft substrates is also a challenge.^[^ ^22^, ^23^ ^]^ At present, most electronic devices on the skin are realized by welding rigid electrical components with stretchable conductors to achieve stretchability.^[^ ^24^, ^25^, ^26^, ^27^, ^28^, ^29^ ^]^ When the stretchable epidermal electronic devices are deformed, electrical failures usually occur at the interfaces between the stretchable conductors and rigid electronic components (soft–rigid connections) due to the stress concentration. To obtain a reliable soft–rigid connection in deformations, researchers usually use solders,^[^ ^30^, ^31^ ^]^ conductive composites,^[^ ^32^, ^33^ ^]^ or vapors^[^ ^34^ ^]^ to reinforce the soft–rigid interfaces. However, such strategies usually involve high temperature (for melting the solders or sintering the conductive composites) or organic solvents, which make in situ connections impossible on some delicate surfaces such as human tissues and biodegradable polymers, because high temperatures or organic solvents can cause irreversible damage to those surfaces.

**Figure 1:**
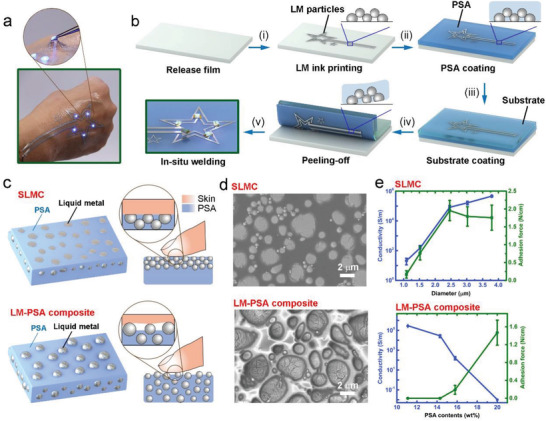
Schematic illustrations and optical images of the SLMC. a) Optical image of a tattoo enabled by the SLMC. Inset: The light‐emitting diode (LED) remains lit even under the action of external force away from the skin. b) The peeling‐off strategy for fabricating the SLMC. (i) printing liquid metal ink on the release film, (ii) coating PSA layer on the liquid metals, (iii) coating substrate layer on the PSA layer, (iv) peeling off the release layer from the PSA layer, (v) elding electronics on the SLMC. Inset: Structure of the liquid metal particles during fabrication. c) Schematic illustrations of the structure of the SLMC and the LM‐PSA composites. d) SEM image of the SLMC and the LM‐PSA composites. e) Electrical conductivity and adhesion force of the SLMC versus the diameter of liquid metal particles (top). Conductivity and adhesion force of the LM‐PSA composites versus PSA contents within the composites (bottom). The sample size was *n* = 12 for each group. The data are presented as the means ± SD.

Here, to overcome those connection difficulties and achieve biocompatible welding on the skin, we develop intrinsically sticky liquid metal conductors (SLMC) that can adhere firmly to different surfaces and form electrical connections. The SLMC is composed of liquid metals and pressure‐sensitive adhesive (PSA) (commercially available polymers prepared from acrylate monomers). The SLMC has a surface‐enriched structure, where the liquid metals enriched on the surface are responsible for forming electrical connections, while the PSA matrix serves to adhere to different surfaces. Thus, once SLMC comes into contact with rigid electronics or soft tissues, it can instantly form both electrical and mechanical connections without involving high temperature and organic solvents that may damage human tissues. The connections achieved by the SLMC are reliable, and these connections will not fail or detach under large deformations such as bending and stretching (**Figure** **1**a). The SLMC is conductive, stretchable, and can be printed into arbitrary 2D patterns. Compared with other conductors with self‐adhesiveness (Table S1, Supporting Information), the SLMC has metal‐grade electrical conductivity (4–6 orders of magnitude higher than hydrogels), hydrogel‐grade stretchability (>900%), and self‐adhesiveness. Moreover, it will not dry out after prolonged air exposure. The electrical conductivity and adhesion of the SLMC will not degrade after exposure to a 60 °C oven for 7 days. Based on the SLMC, we realized the in situ welding of rigid electronics and soft electronics on the skin to form firm connections. To demonstrate that the SLMC can achieve in situ welding of different electronics on the skin, we fabricated a conformal strain sensor circuit that can monitor the movements of the wrist in real‐time. We also realized sticky electrodes that conform to the skin even in deformations for the electrophysiological measurement.

## 2 Results and Discussion

We adopt the peeling‐off strategy (Figure 1b; Figure S1, Supporting Information) to obtain the SLMC with the surface‐enriched structure. We sonicate the Ga/In alloy (80 wt% Ga and 20 wt% In) in decanol to fabricate the liquid metal ink (liquid metal particles dispersing in the solvent of decanol). We use screen‐printing techniques to print the liquid metal ink on a release film (Figure 1b‐i). The release film is a polyethylene terephthalate membrane coated with a thin layer of silicone oil, which can achieve easy detachment with PSA. After printing and evaporating the solvent, liquid metal particles will come into contact and stack with each other on the release film (Figure S2a, Supporting Information). We cast the PSA solutions onto the stacked particles (Figure 1b‐ii), and the solution will penetrate into the gaps between the particles, but the liquid metal particles immersed in the solution will not be separated by floating because the liquid metal particles have larger density than the PSA solution (Figure S1, Supporting Information). After the PSA is cured, we can obtain the surface‐enriched structure on the bottom. The PSA layer is very thin (about 15 µm) and cannot be peeled off without the support of a substrate. So, we need to add a substrate layer to the PSA layer (Figure 1b‐iii). The substrate layer can be any soft material (polyurethane, ethylene‐vinyl acetate, skin, etc.) that can adhere to PSA. Next, we peel off the PSA from the release film (Figure 1b‐iv) to obtain the surface‐enriched structure (Figure 1c (top),d). To activate the liquid metal particles, we applied biaxial strains (about 50%) on the liquid metal printed substrate to break the oxide layer of liquid metal particles and form conductive paths between particles.^[^ ^24^ ^]^ Finally, we obtain the sticky, printable, and conductive SLMC that can achieve an instant connection with delicate surfaces by pressure alone, without involving high temperatures, toxic solvents, or conditions not compatible with human bodies.

To demonstrate the benefits of the peeling‐off strategy for fabricating SLMC, we compared SLMC and the composites of liquid metal and PSA (LM‐PSA composite). To fabricate the LM‐PSA composite, we diluted the PSA with dipropylene glycol methyl ether, and sonicated it with the liquid metals to obtain a paste. We can get LM‐PSA composites after printing the paste. The SLMC and the LM‐PSA composites have different performances due to the different microstructures.

The SLMC can firmly adhere to different surfaces and maintain both high electrical conductivity and adhesion due to the surface‐enriched structure. In contrast, the LM‐PSA composite cannot simultaneously achieve high electrical conductivity and self‐adhesiveness.

SLMC has a surface‐enriched structure, which enables it to have excellent electrical conductivity. No matter how thick the PSA's matrix is, the liquid metal particles will concentrate on the surface of the PSA at a depth of 10 microns to form a conductive network (Figure 1c (top); Figure S3, Supporting Information). As a result, the electrical conductivity of the SLMC is independent of the content of PSA. But the electrical conductivity increases with the diameter of the liquid metal particles (Figure 1e (top)). The surface enriched liquid metal conductive networks endow the SLMC with excellent electrical conductivity. As a comparison, the LM‐PSA composites have a uniform liquid metal distribution. With the increase of the PSA content in the composite, the acrylate adhesive polymers will gradually isolate the contacts between liquid metal particles (Figure S4, Supporting Information). Thus, the electrical conductivity of the composite will drop sharply. To obtain LM‐PSA composites with excellent electrical conductivity, the PSA content in the composite should not exceed about 16 wt%. However, low PSA content results in low self‐adhesiveness of the composite. Therefore, the LM‐PSA composite cannot simultaneously achieve high electrical conductivity and self‐adhesiveness (Figure 1e (bottom)).

The surface‐enriched structure also endows the SLMC with excellent self‐adhesiveness. Thus, the SLMC can have excellent electrical conductivity and self‐adhesiveness at the same time. The adhesiveness of the SLMC comes from the PSA, which are acrylate polymers that can form a bond to the adherend simply by the application of light pressure. The PSAs we applied here are a group of commercially available polymers prepared from acrylate monomers. PSA solutions contain acrylic monomers, including 2‐ethylhexyl acrylate, butyl acrylate, ethyl acrylate, and iso‐octyl acrylate. After evaporation of the solvents, acrylic monomers were polymerized to produce an acrylic pressure‐sensitive polymer. These polymers are soft, transparent, and sticky. The combination of the liquid metal and PSAs is not achieved by simply mixing. Instead, we adopt the peeling‐off strategy to obtain the SLMC with a surface‐enriched structure. The SLMC surface consists of liquid metal particles and PSA. When the SLMC is in contact with other surfaces like the skin, the PSA and liquid metals on the surface will simultaneously come into contact with the skin because the surface of the SLMC is flat and has no protrusions (Figure 1c (top),d (top)). As a result, the liquid metals on the surface are responsible for forming electrical connections, while the PSA serves to adhere to different surfaces. On the contrary, the surface of the LM‐PSA composite exhibits an embedded structure, and the liquid metal particles are embedded in the PSA matrix (Figure 1c (bottom),d (bottom)). When the PSA content is low (<16 wt%), the composite presents excellent electrical conductivity but poor self‐adhesiveness. That is because the surface of the LM‐PSA composite is not flat, and the liquid metals protrude on the PSA matrix. When the LM‐PSA composite comes into contact with the skin, the protruding liquid metals will contact the skin, while it is difficult for the PSA matrix to reach the skin, thus resulting in poor adhesion of the composite. When the PSA content is high (>16 wt%), the composite presents good self‐adhesiveness but becomes an insulator (Figure 1e (bottom)). That is because PSA takes up most of the surface area, and the liquid metals are isolated by the PSA (Figure S4, Supporting Information). As a result, the SLMC can have high electrical conductivity and self‐adhesiveness simultaneously, while the LM‐PSA composites cannot have both properties.

**Figure 2:**
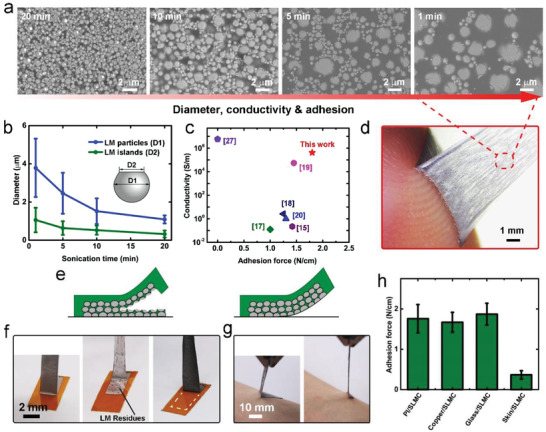
Characterization of the SLMC. a) The SEM characterization of the SLMC composed of liquid metal particles with different diameters. b) Diameter of the liquid metal particles and liquid‐metal islands versus sonication time within the SLMC. The sample size was *n* = 6 for each group. The data are presented as the means ± SD. c) A comparison of electrical conductivity and adhesion force of reported sticky conductors. d) The SLMC can adhere firmly to the skin. e) Schematic illustrations of the liquid metal residues in the substrate after peeling off. f) SLMC with high adhesion will leave liquid metal residues after peeling off, while SLMC with low adhesion will leave no residue. g) No liquid metal residue will be left on the skin after removing the SLMC from the skin. h) The adhesion force between SLMC and different substrates. The sample size was *n* = 12 for each group. The data are presented as the means ± SD.

We can control the properties of the SLMC by adjusting the size of the liquid metal particles (**Figures** 1e,**2**a). The diameter of the liquid metal particles after sonication and the diameter of liquid metal islands on the SLMC surface depend on the sonication time when fabricating the liquid metal ink. With the increase of the sonication time, the diameter of liquid metal particles in ink will decrease, so the diameter of the liquid metal islands will decrease (Figure 2b). The diameter of the liquid metal particles determines the electrical conductivity and self‐adhesiveness of the SLMC. As the diameter of the liquid metal particles decreases, the electrical conductivity of the SLMC will drop sharply (Figure 1e), because liquid‐metal particles with smaller diameters require larger stress to break,^[^ ^24^ ^]^ which will lead to a sharp drop in the conductive paths between liquid metal particles.

The adhesion force of the SLMC can also be adjusted by the diameter of the liquid metal particles. The adhesion force between the PSA and the polyimide (PI) is about 5.1 N cm^−1^ (PSA‐PI, 90° peel adhesion on PI). When the PSA is made into SLMC, the adhesion force (SLMC‐PI) drops below 2.0 N cm^−1^ (Figure 1e). We assumed that a larger proportion of the PSA area on the SLMC surface should have greater adhesiveness, and reducing the PSA area on the SLMC surface leads to a decrease in self‐adhesiveness. As the diameter of the liquid metal particles increase, the proportion of PSA area on the SLMC surface will also increase (Figure S5, Supporting Information), which indicates that the adhesiveness will also increase. However, the assumption that adhesiveness increases with diameter is only satisfied below 2.5 µm (Figure 1e (top); Figure S5, Supporting Information). When the diameter is greater than 2.5 µm, the adhesiveness no longer increases with the diameter. So, according to our assumption, the SLMC should have greater adhesion than the measured value when the diameter is larger than about 2.5 µm.

We analyze the reasons for the inconsistency when the diameter is greater than 2.5 µm. The surface‐enriched structure composed of PSA framework and liquid metal filler is fragile because the liquid metal filler does not contribute to the breaking strength of the structure. Thus, during the test of adhesiveness, the surface‐enriched structure will be torn apart from the middle (Figure 2e (left)) at the critical value (about 1.6 N cm^−1^) before reaching the assumed adhesion force, leaving liquid metal residues on the adherend (Figure 2f (middle)). Thus, the adhesion force of the SLMC is lower than we expected. By contrast, when the diameter of the liquid metal particles in the SLMC is less than about 2.5 µm, the adhesion force of the SLMC will decrease with the diameter as expected. And after peeling off the SLMC from the bonding object, the surface‐enriched structure will not be broken, and no liquid metal residue will remain (Figure 2e (right),f (right)).

We can obtain SLMC with both excellent electrical conductivity and self‐adhesiveness by adjusting the size to about 3.8 µm. The continued increase in size will reduce the accuracy of printing. Compared to other intrinsically sticky conductors such as conductive polymers and conductive hydrogels, our SLMC has comparable self‐adhesiveness. However, the electrical conductivity of SLMC is 4–6 orders of magnitude higher than that of hydrogels (SLMC: ≈417 000 S m^−1^; hydrogel: 0.1–10 S m^−1^) (Figure 2c; Table S1, Supporting Information).

We measure the adhesion force of the SLMC to different materials. The adhesion force of the SLMC to the skin (SLMC‐skin, about 0.36 N cm^−1^) is much lower than the adhesion to PI, copper, and glass (Figure 2h). Also, the adhesion force is lower than the critical value, so no liquid metal residues will remain on the skin after the SLMC is peeled off from the skin (Figure 2d,g).

To increase the adhesion force of the SLMC to the skin as well as the biocompatibility, we use different commercially available PSAs to fabricate the SLMC. Those commercial PSAs (WD‐2000, Ghost Bond, DAVLYN, Ultra Hold) have good biocompatibility with human skin and are designed for attaching wigs to the scalp. The adhesion force of SLMC made of different PSAs to the skin is significantly different, which suggests that it is feasible to adjust the performance of the SLMC by improving the properties of PSA (Figure S6, Supporting Information). Adhesion to wet and dynamic surfaces, such as wet skin, is important in many fields but has proven to be extremely challenging. The PSAs we adopt in this paper cannot be used in wet environments. We tested the adhesiveness using the 90° peel test between the SLMC electrodes and the wet skin, and the adhesion was too small to measure. In the future, we believe that by adopting PSAs that can adhere to wet surfaces, the SLMC can directly adhere to the surfaces of living organs such as the heart and bladder, which have great potential in implantable applications.

**Figure 3:**
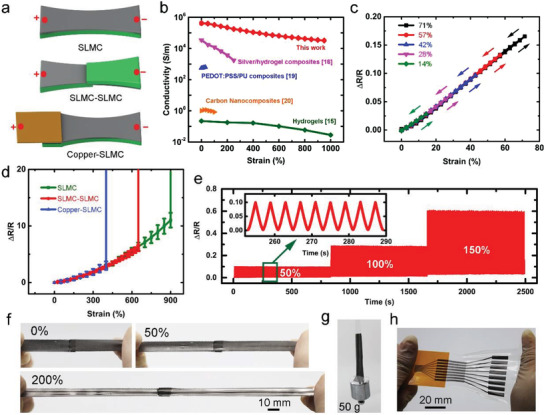
Mechanical–electrical test of the SLMC and connections enabled by the SLMC. a) Schematic illustrations of samples in the mechanical**–**electrical test. b) Comparison of electrical conductivity of the SLMC under strain with other sticky conductors. c) Δ*R*/*R* of the SLMC versus tensile strains in the stretching/releasing test. d) Δ*R*/*R* of the SLMC and connections enabled by the SLMC versus tensile strains. e) Real‐time monitoring of the resistance change of the SLMC–copper connection by stretching the SLMC–copper connection from a strain of 0 to a strain of 50%, 100%, and 150% for about 200 cycles. f) The SLMC–SLMC connection can be stretched to different strains. g) The SLMC with a width of 1 cm can be tightly attached to a steel weight and bear a weight of 50 g. h) The SLMC can be stretchable interfaces between the PCB and stretchable electronics.

We test the stretchability and repeatability of the SLMC and the connections enabled by the SLMC (**Figure** **3**a). The SLMC has high electrical conductivity (≈417 000 S m^−1^) at a strain of 0, and it can be stretched to a strain of 950% and remains conductive (≈ 32 200 S m^−1^) (Figure 3b). Compared with other sticky conductors reported, the SLMC is at least two orders of magnitude higher in electrical conductivity and stretchability (Figure 3b). Compared with typical conductors in stretchable electronics, the SLMC has comparable conductivity and stretchability (Table S1, Supporting Information). The typical conductors contain conductive hydrogels,^[^ ^15^, ^16^ ^]^ composites of carbon nanomaterial,^[^ ^32^ ^]^ and metal nanomaterials.^[^ ^33^, ^35^ ^]^ The conductive hydrogels and composites of carbon nanomaterial usually have poor electrical conductivity (10^−1^–10^3^ S m^−1^). Those conductors have potential as sensors such as strain sensors and electrodes, but they are not suitable as interconnects for stretchable electronics because their electrical conductivity is several orders of magnitude lower than that of metals (about 10^7^ S m^−1^). The metal nanomaterials usually have both excellent electrical conductivity (10^4^–10^6^ S m^−1^) and stretchability (400–1000%); they can be used both as sensors and interconnects for stretchable electronics. The electrical conductivity and stretchability of our SLMC are very close to those of metal nanomaterials. The SLMC also has excellent electrical repeatability. In the stretching/releasing test (strain: 0–14%–0–28%–0–42%–0–57%–0–71%–0), the strain–resistant curve during stretching and releasing almost overlaps, and no hysteresis can be observed in the stress–strain cycle (Figure 3c). After the stretching/releasing test, the resistance variation is less than 0.75% (Figure S7, Supporting Information), suggesting that the SLMC also has great potential as a strain sensor. We adhere the SLMC to another SLMC to form a soft–soft connection and adhere the SLMC to copper to form a soft–rigid connection. To characterize the reliability of those connections, we stretch both ends of the connection and measure the resistance change at both ends, shown in Figure 3a. The three forms of stretching in Figure 3a have a similar trend of resistance changes (Figure 3d). Compared to the SLMC, which breaks at a strain of 950%, the SLMC–SLMC connection breaks at about 650%, while the SLMC–copper connection breaks at about 400%. The SLMC–copper connection also shows excellent repeatability when stretched to a strain of 50%, 100%, and 150% for about 200 cycles, respectively (Figure 3e), and the resistance variation is less than 4% after the cycle test. Therefore, the SLMC combines the advantages of metals and hydrogels. It has metal‐grade electrical conductivity, hydrogel‐grade stretchability, and self‐adhesiveness. The SLMC can directly adhere to different surfaces (human skin, metals) by gentle pressure and form both electrical and mechanical connections. These connections will not fail even under large deformations (Figure 3d,f,g). In addition, the SLMC is printable, which can also be printed as a stretchable interface between the printed circuit board (PCB) and stretchable electronics (Figure 3h). However, the connection between the SLMC and the PCB is irreversible because after peeling the SLMC from the PCB, the surface of the SLMC will remain on the PCB due to the high adhesion (Figure S8, Supporting Information), making the SLMC lose its adhesion.

**Figure 4:**
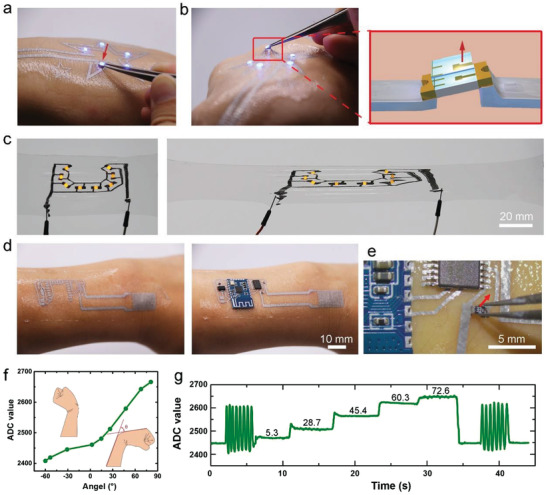
In situ welding of rigid electronics on the SLMC. a) The LED can form both electrical and mechanical connections when in contact with the SLMC. b) The connection between the LED and the SLMC is robust, and the LED remains lit even under the action of external force away from the skin. c) In situ welding of LEDs on the SLMC based on the polyurethane film. The LEDs connected to the SLMC circuit remain lit when stretched. d) In situ welding of electronic components to the SLMC on the skin to form a circuit for monitoring the motion of the wrist. e) The electronic components can adhere tightly to SLMC on the tattoo and will not fall under force away from the skin. f) The analog to digital conversion (ADC) value of the electronic tattoo versus angle of the wrist. g) Real‐time monitoring of the wrist angle using the electronic tattoo.

Based on the SLMC, we can fabricate electronic tattoos on the skin and achieve in situ welding of electronics on the skin. We use a transfer method to make a thin SLMC electronics tattoo (about 28 µm) on the skin and use a tweezer to connect LEDs to the SLMC wires. The LED lights up as soon as it touches the SLMC (**Figure** **4**a; Movie S1, Supporting Information), which suggests that a stable electrical connection is formed upon contact. We use the tweezer to apply an external force away from the skin to the connected LED, and the LED works normally and deforms together with the skin (Figure 4b; Movie S2, Supporting Information), indicating that the LEDs have formed both electrical and mechanical connections with the SLMC (Figure 4b).Despite the fact that we wave our hands or bend our wrists quickly, the connected LED will not fall off, and all the LEDs remain lit.

Based on the SLMC, we can also achieve soft–rigid connections in stretchable circuits. We use the SLMC to fabricate a stretchable LED circuit on the soft polyurethane substrate. Once contacted, the LEDs will form instant connections with the SLMC interconnects. After the connection, we encapsulate the SLMC by spin coating a layer of polyurethane. The LED circuit can still work after being stretched to 100% strain, demonstrating the robustness of the SLMC‐LED (soft–rigid) connections to extreme deformations (Figure 4c).

The reported connection strategies in stretchable circuits usually require a high temperature to melt the solder or a long time to evaporate the organic solvent in conductive pastes. Both high temperature and organic solvent can cause damage to the skin. In contrast, our SLMC can form an instant connection with rigid electronics once contacted with electronics, without heating, solvents, or additional time.

Based on the SLMC and the connection strategy, we fabricated an electronic tattoo that can monitor the motion of the wrist in real‐time (Figure 4d; Movie S3, Supporting Information). This electronic tattoo contains a microcontroller, a voltage divider resistance, a Bluetooth module for sending the signals to the computer, and an SLMC strain sensor (Figure S9, Supporting Information). The layout design of the electronic tattoo is shown in Figure S10, Supporting Information. The SLMC strain sensor with a serpentine structure has the same fabrication process as the SLMC wires, while other electronics in the tattoo are connected to the tattoo by instant contact. Those connected electronics will not fall off, even if we try to pull them away from the skin with tweezers (Figure 4e). When we bend the wrist, the SLMC strain sensor at the wrist will be stretched, and the resistance will increase. We measured the relationship between the wrist angle and the output value (ADC value) of the electronic tattoo shown in Figure 4f. We have realized real‐time monitoring of the wrist angle by wearing the electronic tattoo (Figure 4g). The output signals have good repeatability when bending the wrist with high frequency. The output signals from the strain sensor are stable when the wrist remains stationary and will not decay with time (Figure 4g).

**Figure 5:**
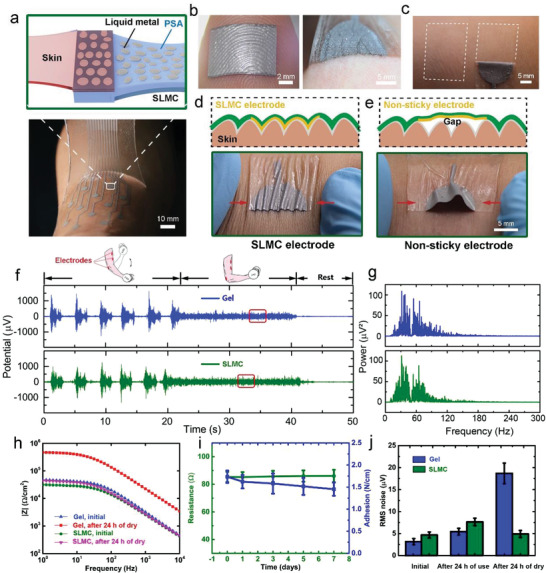
In situ welding of SLMC electrodes on the skin. a) The SLMC can adhere to the soft skin (top). An electrode array based on the SLMC (bottom). b) The SLMC is conformal, stretchable, sticky, and printable. It can be embedded into the fingerprint and deform with the skin. c) The SLMC causes no skin irritation and visible redness after the use of 24 h. Schematic illustration and optical image of the d) sticky electrode and e) non‐sticky electrode attached to the squeezed skin. f) Comparison of EMG signals using SLMC electrodes and commercial gel electrodes. g) Power spectrogram of the EMG signal recorded using the SLMC electrode in the isometric contraction period. h) The impedance spectra of the commercial gel electrode and SLMC electrode on the skin in the initial state and after 24 h of drying. i) Resistance and adhesion force (SLMC‐PI) of the SLMC versus exposure time in a dry oven at 60 °C. The sample size was *n* = 12 for each group. The data are presented as the means ± SD. j) The RMS noise of the commercial gel electrodes and SLMC electrodes during EMG recording in the initial state, after 24 h of use, and after 24 h of drying. The sample size was *n* = 12 for each group. The data are presented as the means ± SD.

The SLMC can be adhered to the skin and used as soft electrodes for epidermal bio‐potential monitoring (**Figure** **5**a). The SLMC has excellent conformability with the skin, and it can be embedded into the fingerprint when its thickness is less than 30 µm (Figure 5b). The SLMC electrodes have excellent biocompatibility with the skin because both the liquid metal and PSA (commercial PSA for wig) constituting the electrode are non‐toxic. To prove that the SLMC electrodes are non‐toxic, we attached electrodes to the skin of the upper arm for 24 h. After removing the electrodes, the surface of the skin remains clean (no visible residue), and only some wrinkles appear on the skin. No itchiness, irritation, or other feelings of discomfort occurred during use (Figure 5c). The reason for the formation of wrinkles is that when the elbow is bent, the skin at the upper arm will contract, and the electrode will deform simultaneously with the skin. However, the electrode cannot contract. As a result, the electrode and the skin will form wrinkles together. We used the SLMC with different thicknesses to test the moisture permeability. The SLMC provides excellent moisture permeability when the thickness of SLMC is less than about 60 µm (Figure S11, Supporting Information). In this case, the amount of moisture permeating the SLMC exceeds the moisture lost from the skin through insensible perspiration (≈600 g m^−2^day^−1^).^[^ ^36^ ^]^ SLMC is printable and can be printed as an electrode array, which has the potential for high‐resolution electromyography (EMG) detection (Figure 5a). Due to the good adhesion and compliance, the SLMC electrodes can adhere firmly to the skin and deform with the skin. Even if we stretch the skin or squeeze the skin, the electrode will not detach from the skin (Figure 5d). Thus, there will be no gap at the electrode–skin interface to cause noise in the bio‐potential measurement. On the contrary, although the non‐adhesive stretchable electrode can attach to the skin, the electrodes cannot be deformed simultaneously with the skin. When the skin is squeezed, it will detach from the skin, creating a huge gap at the electrode–skin interface (Figure 5e).

We use the SLMC electrodes to monitor the surface electromyography of the biceps when lifting the dumbbell. Two SLMC electrodes are placed on the biceps of a volunteer, and one SLMC electrode is placed on the elbow (electrically unrelated tissue) (Figure 5f (top)). The volunteer was required to raise the dumbbell with one hand and carry out concentric and eccentric contraction exercises of the biceps for five cycles, and then sustain an isometric contraction at 90° of flexion for about 20 s. As a control, we attached three commercial hydrogel electrodes (3M Red Dots) to the same positions as the SLMC electrodes and repeated the above measurement process. The EMG signal using the SLMC electrodes is comparable to that with the hydrogel electrode (Figure 5f). We analyzed the power spectrogram of the EMC signal in the isometric contraction period (Figure 5g). The signals collected by the SLMC electrode and the hydrogel electrode have the same frequency distribution, and the frequency is located between 10 and 250 Hz, which matches the frequency range of the EMG signals.

Compared to commercial hydrogel electrodes, our SLMC electrode is resistant to drying, such that it will not evaporate and dry out like a hydrogel electrode. The SLMC electrode exhibits a lower electrode–skin impedance than that of the hydrogel electrode in the frequency range of 1–10^4 ^Hz (Figure 5h), which can be attributed to the high electrical conductivity of the SLMC. We exposed the SLMC electrodes and the hydrogel electrode in a drying oven at 37 °C. After 24 h of drying, the SLMC electrode still maintained adhesion, and the electrode–skin impedance remained almost unchanged. On the contrary, the hydrogel electrode becomes dry and hard due to evaporation, loses its adhesion to the skin (Figure S12, Supporting Information), and significantly increases the electrode–skin impedance. To further prove the drying resistance of the SLMC, we exposed the SLMC to dry air at 60 °C for 1 week. After 1 week of drying, the SLMC still maintained high adhesion, and the electrical resistance remained almost unchanged (Figure 5i).

We carried out the root‐mean‐squared (RMS) analysis to obtain the noise of the EMG signals during the isometric contraction. The RMS noise from the SLMC electrodes is about 4.67 µV, which is comparable to the commercial gel electrodes (3.15 µV) (Figure 5j). It is also much lower than that of other dry electrodes in the literature.^[^ ^19^, ^21^, ^37^ ^]^ After sticking on the biceps for 24 h, the noise of the SLMC increases to 7.65 µV, while the noise of the gel electrode increases to 5.42 µV. After 24 h of exposure in a drying oven, the RMS noise of the gel electrode is increased by 6 times. In contrast, the RMS noise of the SLMC electrode hardly changes, and it can still adhere firmly to the skin. The SLMC is resistant to drying, which makes the SLMC electrode have great potential for long‐term storage and long‐term bio‐potential monitoring.

## 3 Conclusion

In this work, we have achieved conductors with excellent electrical conductivity, stretchability, and adhesion, which can achieve biocompatible in situ welding to delicate surfaces, such as those on human tissues. The traditional welding of electronic devices is achieved by melting solders (usually tin alloys) at high temperatures (>200 °C), which can cause damage to delicate surfaces such as skin. Conductive composites/pastes can be a welding substitute. Those composites/pastes are highly conductive and can form stretchable connections between the electronics and the interconnects. However, those composites/pastes usually contain organic solvents, which are toxic to human tissues. Some composites/pastes also require a high temperature (>100 °C) for sintering to obtain conductivity, which is beyond the temperature range that the human body can withstand. In addition, connections enabled by the composites/pastes are not instant, which usually takes several hours for the solvent in the composite/paste to evaporate. The extra evaporation time makes the in situ welding difficult, as it requires the subject to remain still for several hours.

In contrast, the SLMC can achieve biocompatible in situ welding of electronics on human skin without involving high temperatures or organic solvents. In addition, our SLMC can form an instant connection with electronics as soon as the SLMC contacts with electronics, without requiring subjects to remain immobile for a long time.

Compared with conductive hydrogels with adhesion, our SLMC is printable and drying‐resistant. The SLMC will not dry out after being exposed to air for a week and retain high electrical conductivity and adhesion. The resistance to drying makes the SLMC a great potential for long‐term storage and long‐term healthcare monitoring.

Based on the SLMC, we provide an effective method for establishing ohmic contact between tissue‐compatible soft electronics. The SLMC can serve as soft–rigid interfaces in stretchable electronics. Rigid electronics can form both electrical and mechanical connections with the SLMC once in contact. The SLMC can also be used for soft–soft interfaces. The SLMC can adhere firmly to the skin and deform simultaneously with the skin. The SLMC has a high electrical conductivity of about 417 000 S m^−1^. However, its electrical conductivity is still two orders of magnitude lower than that of metals, limiting its application in high‐current circuits or circuits with high accuracy. In the future, we need to improve the conductivity of the SLMC. We can improve the conductivity by increasing the uniformity of the particles and removing small‐sized particles. We can also increase the conductivity of the SLMC by adding nano/microstructured metals. We believe that such nano/microstructured metals can serve as conductive paths between the liquid metal particles.

## 4 Experimental Section

### Fabrication of the SLMC

The fabrication of the SLMC started from fabricating the liquid metal ink. The eutectic Ga/In(75.5 wt% Ga and 24.5 wt% In), Ga/In (80 wt% Ga and 20 wt% In), and Galinstan (68.5 wt% Ga, 21.5 wt% In, and 10.0 wt% Sn) are typical commercially available Ga/In alloys. These alloys are liquid at room temperature and have similar physical properties (electrical conductivity: 3 × 106 S m^−1^; density: ≈6 g cm^−3^; viscosity: 2 × 10^−3^ kg m^−1^s^−1^). Thus, those alloys were substitutes for fabricating the SLMC. 3 g liquid metal (gallium–indium alloy, Hawk, HK3284, 80 wt% Ga and 20 wt% In, China) was added in 1 mL n‐decyl alcohol (98%, MACKLIN, China) and sonicated the liquid metal to obtain the liquid metal ink using a probe sonicator (S450D, Branson, USA). The liquid metal was sonicated for 60 s at an amplitude of 20%. Screen‐printing techniques (screen, 200 mesh) were used to print the liquid metal particles on a release film (007, Huahong Composite Materials Co., Ltd., China) and dried them in an oven (DHG‐9420A, Yiheng Scientific Instrument Co. Ltd., China) at 80 °C for 10 min. PSAs were coated on the liquid metal patterns by spin‐coating (2000 rpm, 20 s, KW‐4A, SETCAS, China) and cured them in an oven at 80 °C for 20 min. The PSAs tested in this research included WD‐2000 (solid content 75 wt%, Wen Ding Co. Ltd., China), Ghost Bond (Pra lab, USA), Davlyn (Davlyn, USA), and UItra Hold (Walker Tape, USA). After the PSA was cured, the PSA could be attached to desirable substrates such as thermoplastic polyurethane membranes (DS3412, 0.05 mm, Tunsing, China) and the skin. To obtain a thinner substrate, the polymer solutions could also be spin‐coated on the PSA at a speed of 2000 rpm for 20 s. The polymer solution used here was 30 g of poly(styrene‐butadiene‐styrene) (30 wt%; average Mw ≈ 140 000; Sigma Aldrich, USA) dissolved in 170 g of di(propylene glycol) methyl ether acetate. After the substrate was cured, the release film was peeled off and the SLMC was stretched to a strain of about 50% to active the SLMC. The SLMC was ready for applications of electrophysiology electrodes and stretchable circuits. To protect the SLMC from abrasion and moistures, the SLMC was usually encapsulated with a layer of polyurethane. Polyurethane (PU, BASF, 9390, Germany) was dissolved in the *N*,*N*‐dimethylformamide to obtain a polyurethane solution with a solids content of 10%. For the encapsulation of the stretchable LED circuit, the polyurethane solution was directly spin coated on the PSA layer at 1000 rpm for 30 s after the connection of the LEDs on the SLMC interconnects. For the encapsulation of the electronic tattoos, the polyurethane film was attached with a thickness of 20 µm to the tattoo after finishing the in situ welding of electronics on the skin. The polyurethane film could adhere firmly to the tattoo due to the self‐adhesiveness of the tattoo.

### Fabrication of the LM‐PSA Composites

The PSA (WD‐2000) was diluted with dipropylene glycol methyl ether (Sigma Aldrich, USA) to obtain PSA with different solid contents (75, 56.25, 50, and 37.5 wt%). 3 g liquid metal was mixed with 1 mL diluted PSA and sonicated (60 s at an amplitude of 20%) the mixer to obtain pastes. The screen printing technique was used to print pastes on the thermoplastic polyurethane membranes. After baking in an oven at 80 °C for 10 min and stretching the polyurethane membrane to a strain of 50%, LM‐PSA composites were obtained.

### Characterization of the SLMC

The scanning electron microscope (SEM, Hitachi, S8220, Japan) was used to characterize the surface of the SLMC and the liquid metal particles. To characterize the cross section of the SLMC, a layer of polyurethane was coated on the SLMC (spin‐coating the polyurethane solution on the SLMC at 1000 rpm for 30 s). The polyurethane‐coat SLMC was treated with liquid nitrogen and the SLMC was embrittled to obtain the cross section. The thickness of the PSA (WD‐2000) was measured by a stylus profiler (Bruker, DektakXT, USA). The adhesion force of the SLMC between different surfaces was measured by the 90° peel test using the universal testing machines (3365, Instron, USA). The electrical property with the deformations of the SLMC (PSA: WD‐2000) was measured by a programmable linear guide slide (FSL_40, FUYU, China) and an electrochemical station (1040C, CHI, China). The slider was used to stretch the SLMC to different strains and measure the resistance of the SLMC in real‐time through the electrochemical station. To characterize the long‐term property of the SLMC, the SLMC samples were exposed in an oven at 60 °C for 7 days. The resistance of the SLMC and adhesion force between the SLMC and the polyimide film were measured every 24 h. In the permeability test, SLMCs with different thicknesses were attached to the opening of plastic dishes (35 mm in diameter) containing pure water, and the water vapor permeability was estimated by measuring the weight loss of water. The measurements were carried out while samples were stored in a chamber at 25 °C temperature and 20% humidity. The weight of SLMC film and water in the dish were tested every 12 h. The biocompatibility of the SLMC (PSA: UItra Hold) was tested by attaching the SLMC to the biceps skin of the authors. This test was approved by the Institutional Ethics Committee (Medical Ethics Committee of Capital Medical University, 2021SY021).

The impedance between the SLMC electrode and the skin was performed with Multi Autolab/M204 potentiostat (Metrohm, Switzerland) within a frequency range from 1 Hz to 10 kHz. A sinusoid stimulation voltage with a root‐mean‐square amplitude of 10 mV (no DC offset) was applied to obtain the impedance curve. Two SLMC electrodes (diameter 16 mm) were attached to the Musculus biceps brachii at a distance of 20 mm, and one reference SLMC electrode was attached to the elbow. As a comparison, the impedance of the commercial electrodes (Red Dot, 3M, USA) was also tested through the same process. Both types of electrodes were circular with a diameter of 16 mm.

### SLMC for the EMG Recording

Two working SLMC electrodes and one SLMC reference electrode were connected to g.HIamp multi‐channel amplifier (G.tek, Austria) for bipolar surface EMG data acquisition with a sampling frequency of 1200 Hz and an analog notch filter at 48–52 Hz. Surface EMG signals were recorded from the Musculus biceps brachii of each subject using a bipolar electrode configuration with an inter‐electrode distance of 20 mm. As a comparison, the commercial electrodes (Red Dot, 3M, USA) were also used to record the surface EMG through the same process.

### Fabrication of the Stretchable LED Circuit

To fabricate the stretchable LED circuit, liquid metal ink was used to print interconnects on the release film. The PSA (WD‐2000) layer was coated on the liquid metal. After the PSA was cured, the polyurethane solution was spin coated on the PSA layer at 1000 rpm for 30 s. After the curing of the polyurethane layer, the release film was peeled off, and interconnects will be transferred from the release film to the cured membrane. The membrane was stretched to make interconnects conductive. A tweezer was used to connect LEDs on the SLMC interconnects. Finally, the polyurethane solution was spin coated on the SLMC to encapsulate the SLMC.

### Fabrication of the Electronic Tattoo for Wrist Monitoring

To fabricate the electronic tattoo, liquid metal ink was used to print interconnects (Figure S10, Supporting Information) on the release film. The PSA (UItra Hold) layer was coated on the liquid metal. After the PSA was cured, the PSA layer was attached to the skin. The SLMC interconnects will remain on the skin after the release film was peeled off. The skin around the SLMC was stretched and rubbed to make it conductive. A tweezer was used to connect different electronic components on the SLMC interconnects. The circuit design of the electronic tattoo for wrist monitoring is shown in Figure S9, Supporting Information. The circuit contained a microcontroller (N76E003AT20, Nuvoton, China), a Bluetooth module (WS8100_01, Wisesun, China), a voltage regulator (HT7533‐1, Holtek, China), and a voltage divider resistance.

### Statistical Analysis

All data are expressed as the means ± standard deviation (SD). The sample size (*n*) for each statistical analysis was chosen as 12 unless otherwise indicated. Origin software (OriginLab) was used to calculate the standard deviation *σ* and mean value *μ* for statistical analysis.

## Conflict of Interest

The authors declare no conflict of interest.

## Author Contributions

L.T. and X.J. conceived and supervised this project. L.T. designed, fabricated, and characterized the SLMC. K.Z. and S.Y. characterized the SLMC electrodes and carried out the EMG test. L.T., S.Y., and X.J. wrote the manuscript. All authors discussed the results and commented on the manuscript.

## Supporting information

Supporting Information

Supplemental Movie 1

Supplemental Movie 2

Supplemental Movie 3

## Data Availability

The data that support the findings of this study are available from the corresponding author upon reasonable request.
