# Supplementary material for: Skin Electronics from Biocompatible In Situ Welding Enabled By Intrinsically Sticky Conductors
Source: Adv Sci (Weinh). 2022 Jun 26;9(23):2202043. doi: 10.1002/advs.202202043 (PMC9376824; doi:10.1002/advs.202202043)
Supplement: Supplementary file 1 — Supporting Information [file ADVS-9-2202043-s002.pdf]

ADVANCED SCIENCE
Open Access

Supporting Information

for *Adv. Sci.*, DOI 10.1002/advs.202202043

Skin Electronics from Biocompatible In Situ Welding Enabled By Intrinsically Sticky Conductors

Lixue Tang, Shuaijian Yang, Kuan Zhang* and Xingyu Jiang**

Supporting Information for

Title: Skin electronics from biocompatible in situ welding enabled by intrinsically sticky conductors

Lixue Tang, Shuaijian Yang, Kuan Zhang* and Xingyu Jiang**

The file includes:

Figure S1 to S12

Figure S1. Schematic illustrations for fabricating the SLMC.

Figure S2: SEM characterization of the liquid metal particles and islands.

Figure S3: SEM characterization of the cross section of the SLMC.

Figure S4: SEM characterization of the LM-PSA composites with different content of PSA.

Figure S5: Adhesion force of the SLMC and ratio of PSA area in SLMC to the total area versus sonication time.

Figure S6: SLMC composed of different PSA has different adhesion.

Figure S7: Stretching/releasing test of the SLMC strain sensor.

Figure S8: Liquid metal residues will remain after remove the SLMC from the rigid conductors.

Figure S9: Circuit design and chip summary for the wrist monitoring electronic tattoo.

Figure S10: Layout designs for the wrist monitoring electronic tattoo.

Figure S11: Moisture permeability test of the SLMC with different thicknesses.

Figure S12: The adhesion force of the commercial gel electrode and SLMC electrode on the skin in the initial state and after 24 hours of drying.

Table S1: Conductivity, stretchability, adhesion, printability of various stretchable conductors.

Other Supporting Material for this manuscript includes the following:

Movies S1: In situ welding of electronics on human skin

Movies S2: The LEDs have formed both electrical and mechanical connections with the SLMC.

Movies S3: The electronic tattoo for movement monitoring

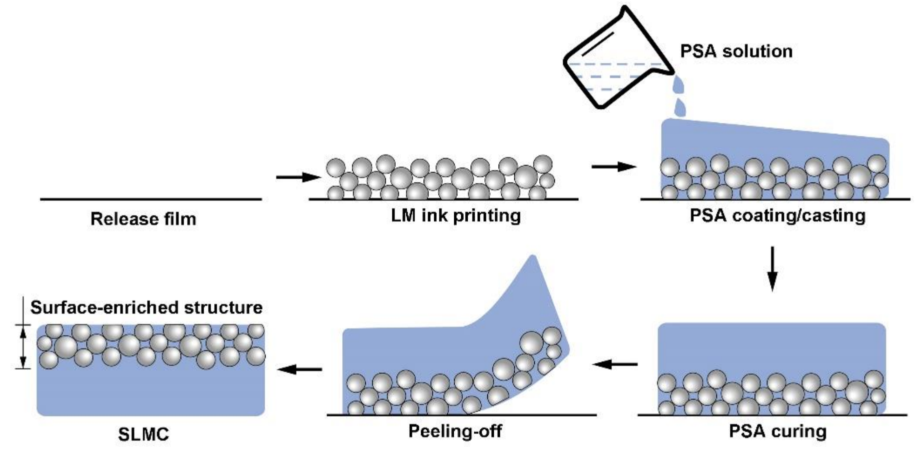

Figure S1. Schematic illustrations for fabricating the SLMC. We adopt the peeling-off strategy to obtain the SLMC with the surface-enriched structure

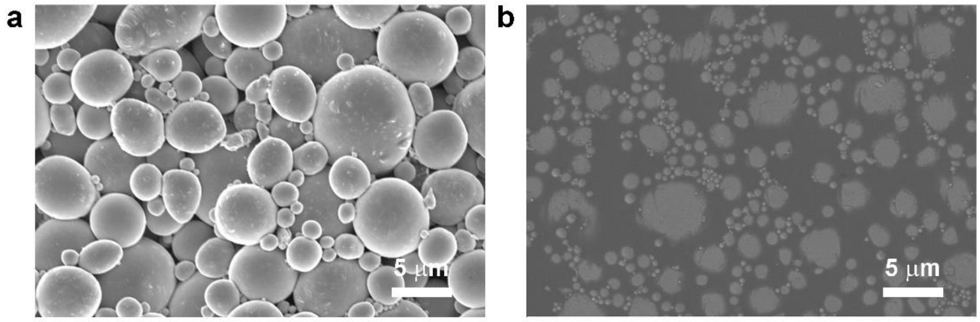

Figure S2. SEM characterization of the liquid metal particles and islands. (a) SEM characterization of the liquid metal particles. (b) SEM characterization of the surface of the SLMC.

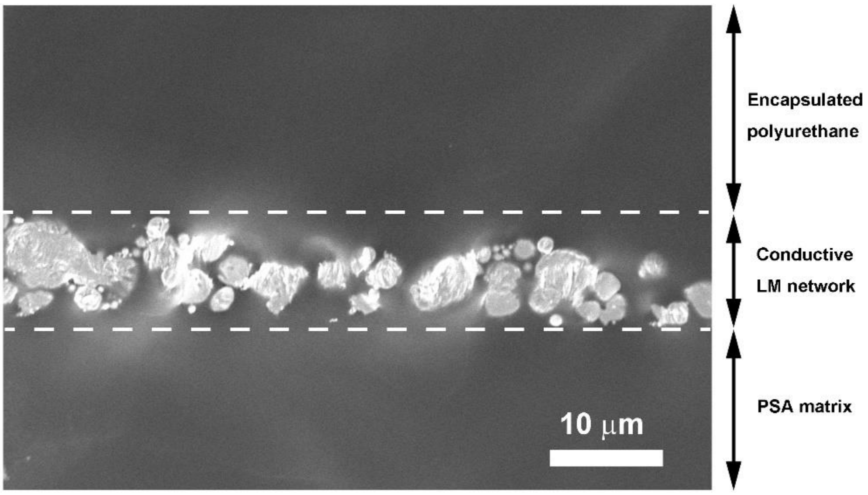

Figure S3. SEM characterization of the cross section of the SLMC. The liquid metal particles will concentrate on the surface of the PSA at a depth of 10 microns to form a conductive network.

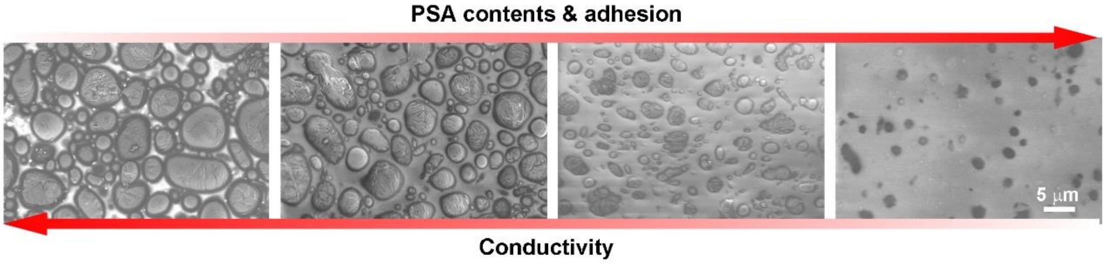

Figure S4. SEM characterization of the LM-PSA composites with different content of PSA. With the increase of the PSA content in the composite, the acrylate polymers will gradually isolate the contacts between liquid metal particles. The PSA content from left to right is 11.1%, 14.3%, 15.8%, and 20%, respectively.

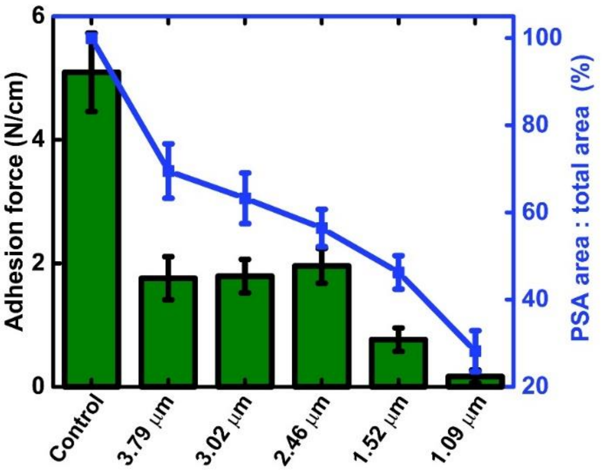

Figure S5. Adhesion force of the SLMC and ratio of PSA area in SLMC to the total area versus diameter of liquid metal particles. Control, the PSA of WD-2000.

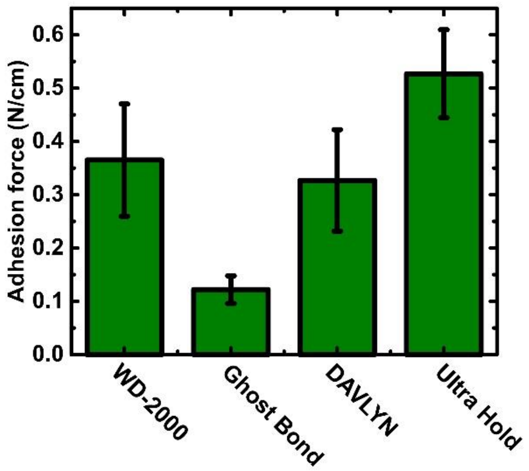

Figure S6. SLMC composed of different PSA has different adhesion. WD-2000, Ghost bond, DAVLYN and Ultra Hold are commercial available pressure-sensitive adhesives

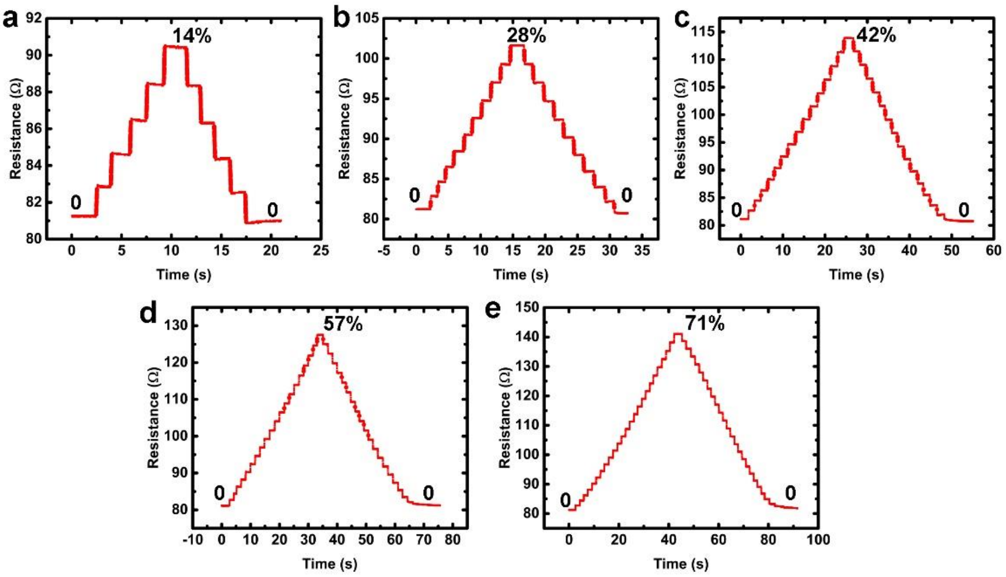

Figure S7. Stretching/releasing test of the SLMC strain sensor. (a) Strain loading method: from 0 to 14% and then back to 0, each strain loading is 2.8%. (b) Strain loading method: from 0 to 28% and then back to 0, each strain loading is 2.8%. (d) Strain loading method: from 0 to 42% and then back to 0, each strain loading is 2.8%. (b) Strain loading method: from 0 to 57% and then back to 0, each strain loading is 2.8%. (e) Strain loading method: from 0 to 71% and then back to 0, each strain loading is 2.8%.

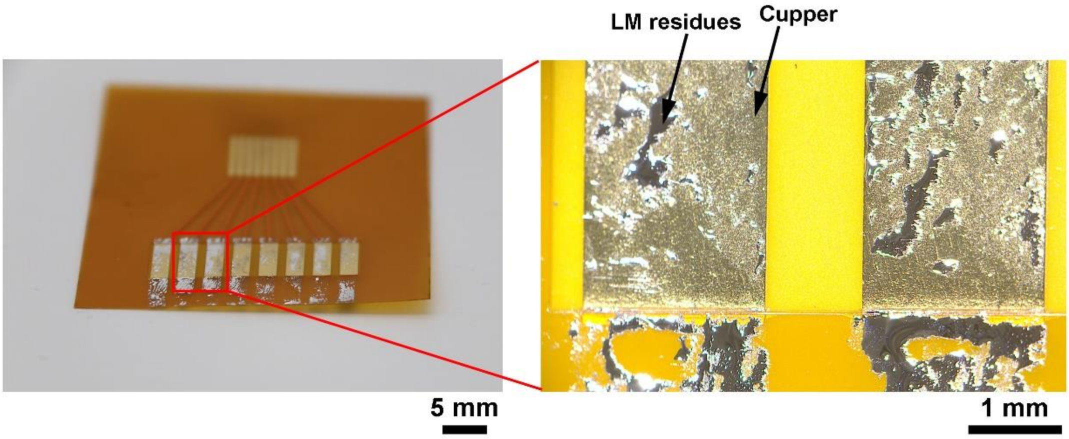

Figure S8. Liquid metal residues will remain after remove the SLMC from the rigid conductors.

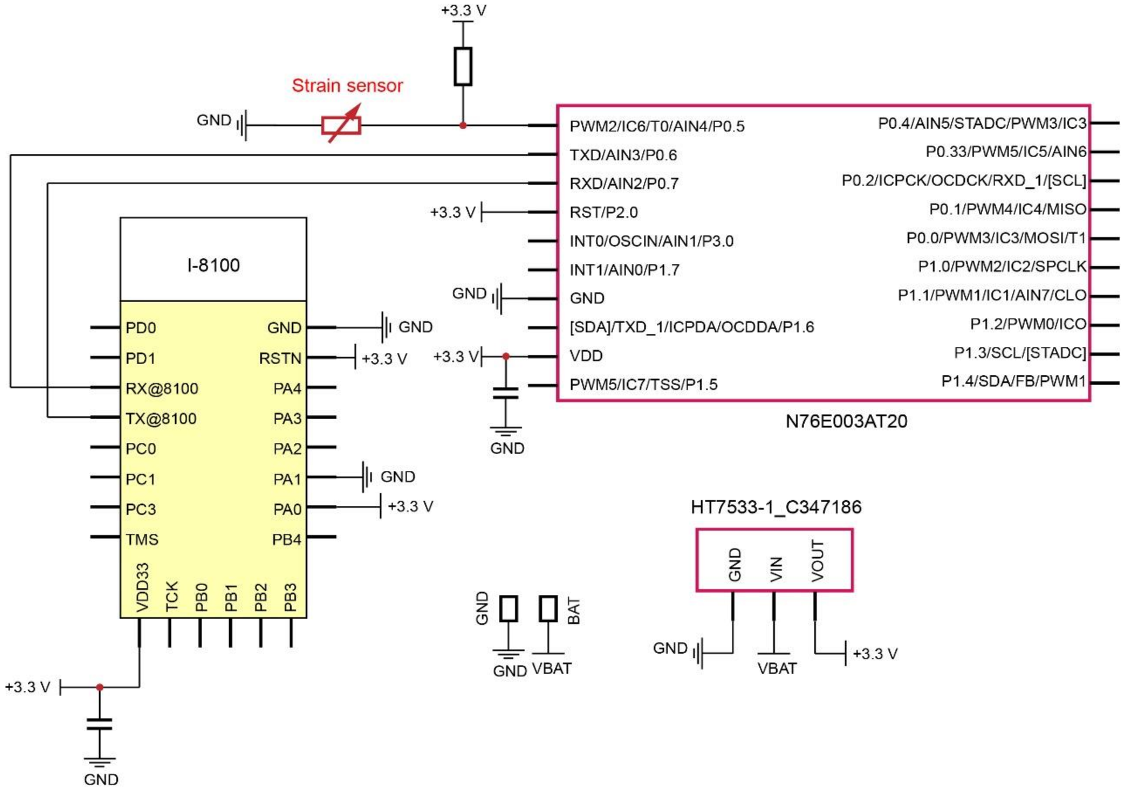

Figure S9. Circuit design and chip summary for the wrist monitoring electronic tattoo.

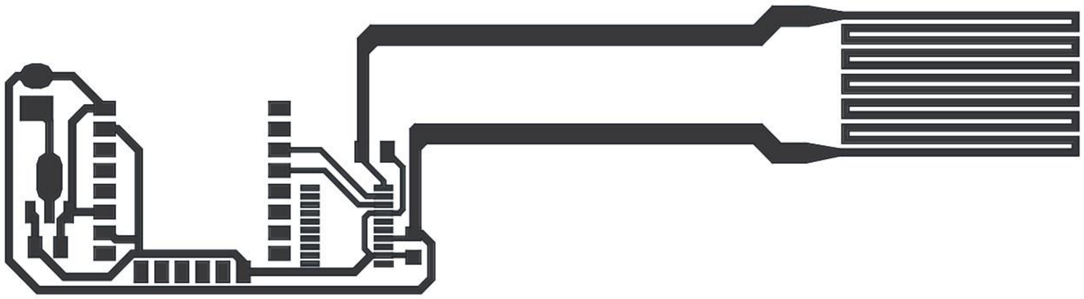

Figure S10. Layout designs for the wrist monitoring electronic tattoo.

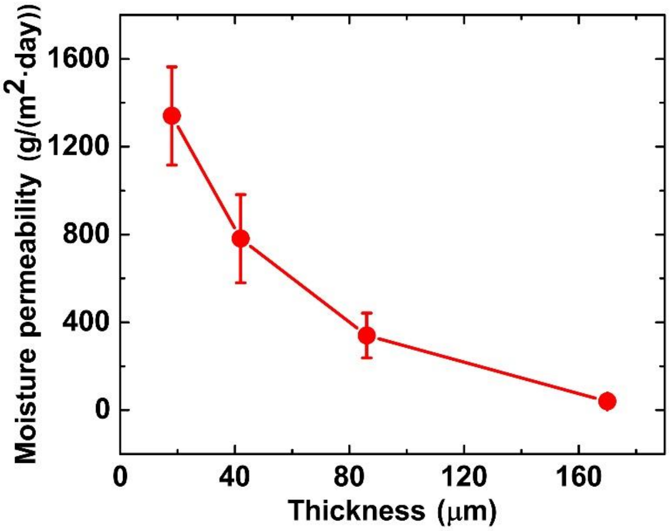

Figure S11. Moisture permeability test of the SLMC with different thicknesses.

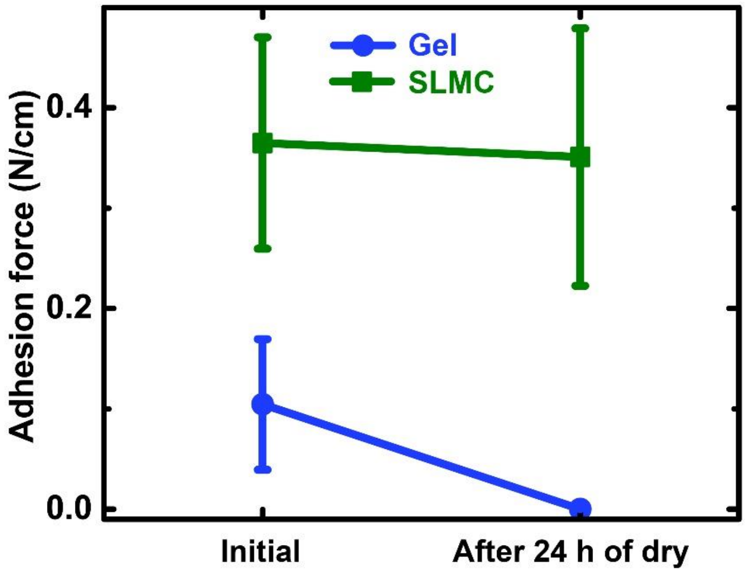

Figure S12. The adhesion force of the commercial gel electrode and SLMC electrode on the skin in the initial state and after 24 hours of drying.

Table S1. Conductivity, stretchability, adhesion, printability of various stretchable conductors.

Ref.	Conductor	Conductivity(S/m)	Stretchability	Adhesion (N/cm)	Printable
This work	**SLMC**	**417000**	**950%**	**1.8**	**yes**
1	PEDOT/PU composite	54500	43%	~1.5	No
2	Sucker-shaped carbon nanocomposites	1	100%	~1.3	No
3	Sucker-shaped PDMS/carbon black composites	80	100%	~5	No
4	Polyacrylic acid Hydrogel	0.126	> 1000%	~1	No
5	Graphene oxide/PVA hydrogel	2.6	>200%	~1.2	No
6	PEDOT/guar slime hydrogel	0.222	> 1000%	~1.4	No
7	Ag nanowire/PDMS	35 Ω/sq	>400%	~0.4	yes
8	Au-Ag/SBS composite	7260000	>840%	<0.01	yes
9	Ag flakes/fluorine rubber composite	73800	>200%	<0.01	yes
10	Liquid metal/polymer composite	1110000	>1000%	<0.01	yes
11	Wavy structured gold	44200000	140%	<0.01	yes
12	CNT/PDMS	10	40%	<0.01	yes

Supplementary References

1. Zhang, L. et al. Fully organic compliant dry electrodes self-adhesive to skin for long-term motion-robust epidermal biopotential monitoring. Nat. Commun. 2020, 11, 4683.
2. Kim, T., Park, J., Sohn, J., Cho, D. & Jeon, S. Bioinspired, highly stretchable, and conductive dry adhesives based on 1D–2D hybrid carbon nanocomposites for all-in-one ECG electrodes. *ACS Nano* 2016, **10**, 4770-4778.
3. Chun, S. et al. Conductive and stretchable adhesive electronics with miniaturized octopus-like suckers against dry/wet skin for biosignal monitoring. *Adv. Funct. Mater.* 2018, **28**, 1805224.
4. Li, G. et al. A stretchable and adhesive ionic conductor based on polyacrylic acid and deep eutectic solvents. npj Flex. Electron. 2021, 5, 23.
5. Deng, J. et al. Electrical bioadhesive interface for bioelectronics. Nat. Mater. 2021, 20, 229–236.
6. Li, S., Wang, L., Zheng, W., Yang, G. & Jiang, X. Rapid Fabrication of Self-Healing, Conductive, and Injectable Gel as Dressings for Healing Wounds in Stretchable Parts of the Body. Adv. Funct. Mater. 2020, 30, 2002370.
7. Kim, J.-H., Kim, S.-R., Kil, H.-J., Kim, Y.-C. & Park, J.-W. Highly conformable, transparent electrodes for epidermal electronics. Nano Lett. 18, 4531-4540 (2018).
8. Choi, S. et al. Highly conductive, stretchable and biocompatible Ag–Au core–sheath nanowire composite for wearable and implantable bioelectronics. Nat. Nanotechnol. 2018, 13, 1048–1056.
9. Matsuhisa, N. et al. Printable elastic conductors with a high conductivity for electronic textile applications. Nat. Commun. 2015, 6, 7461.
10. Tang, L., Mou, L., Zhang, W. & Jiang, X. Large-Scale Fabrication of Highly Elastic Conductors on a Broad Range of Surfaces. ACS Appl. Mater. Interfaces 2019, 11, 7138–7147.
11. S. Xu, Y. Zhang, L. Jia, K. E. Mathewson, K. I. Jang, J. Kim, H. Fu, X. Huang, P. Chava, R. Wang, S. Bhole, L. Wang, Y. J. Na, Y. Guan, M. Flavin, Z. Han, Y. Huang, J. A. Rogers, Science 2014, 344, 70–74.

12. J. Dou, L. Tang, L. Mou, R. Zhang, X. Jiang, Compos. Sci. Technol. 2020, 197, 108237.
